# Supplementary material for: Structure can predict function in the human brain: a graph neural network deep learning model of functional connectivity and centrality based on structural connectivity
Source: Brain Struct Funct. 2021 Oct 11;227(1):331–43. doi: 10.1007/s00429-021-02403-8 (PMC8741721; doi:10.1007/s00429-021-02403-8)
Supplement: Supplementary file 1 — Supplementary file1 (DOCX 5143 KB) [file 429_2021_2403_MOESM1_ESM.docx]

**Supplementary Material**

*
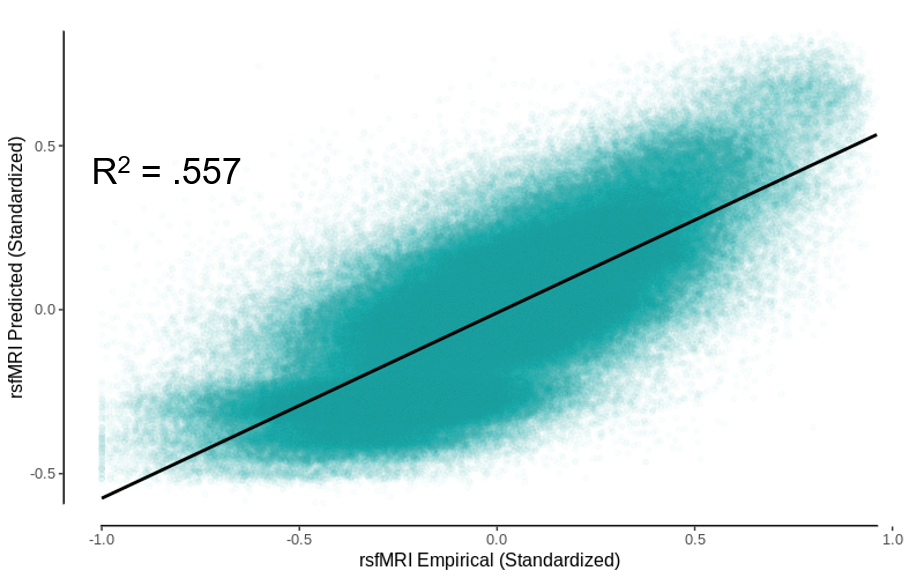
*

*Supplementary Figure 1.* DK atlas non-aggregated predicted rsfMRI functional connectivity as a function of empirical rsfMRI functional connectivity (R^2^ = .557).

*
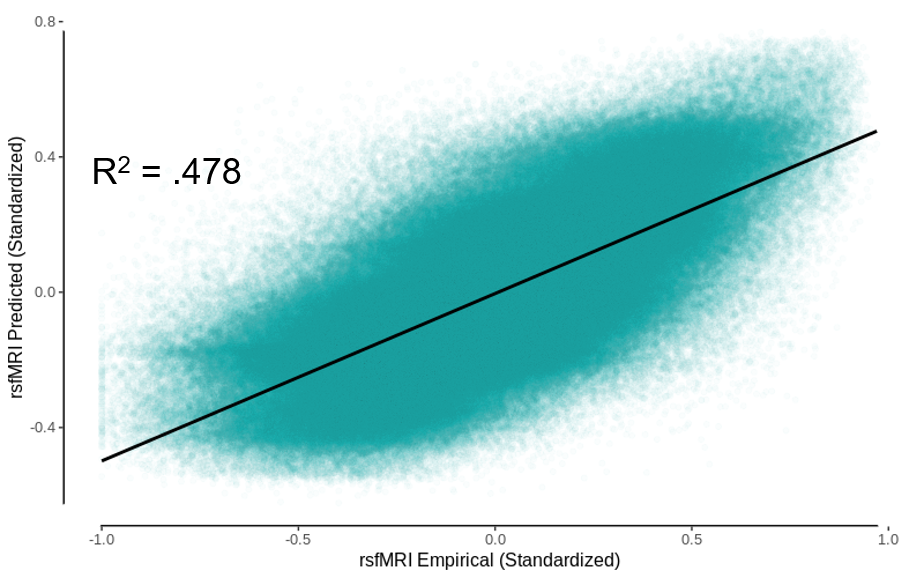
*

*Supplementary Figure 2.* AAL atlas non-aggregated predicted rsfMRI functional connectivity as a function of empirical rsfMRI functional connectivity (R^2^ = .478).


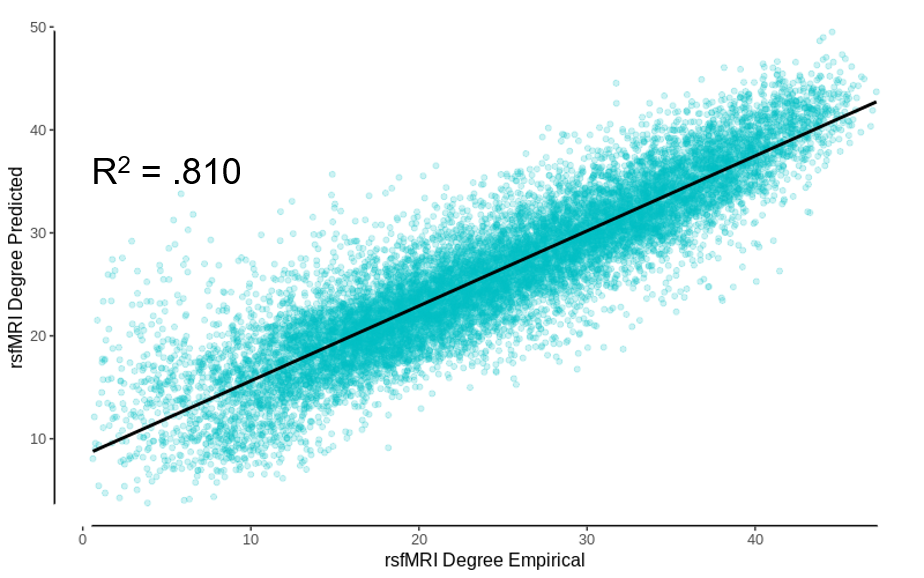


*Supplementary Figure 3*. DK atlas non-aggregated degree centrality measures calculated from the predicted rsfMRI functional connectivity values as a function of empirical rsfMRI functional degree centrality (R^2^ = .810).


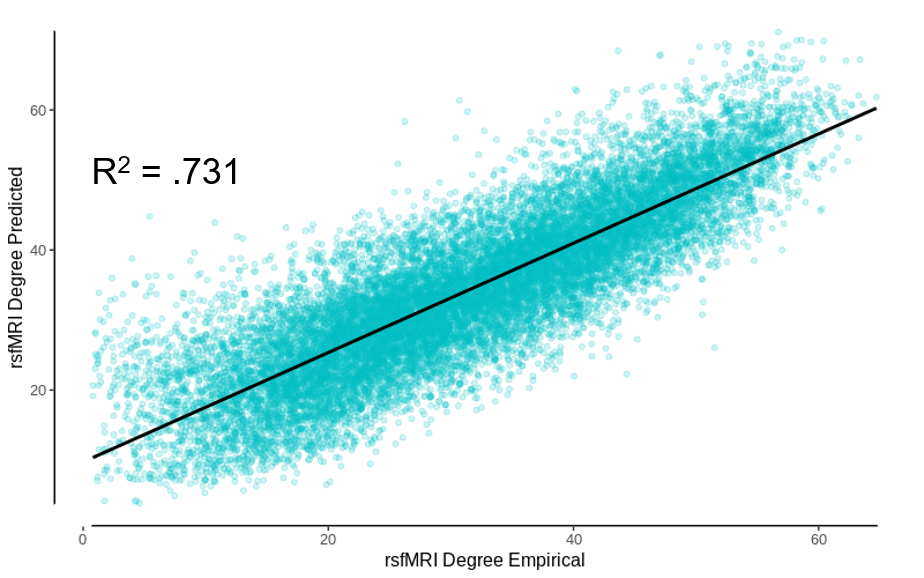


*Supplementary Figure 4*. AAL atlas non-aggregated degree centrality measures calculated from the predicted rsfMRI functional connectivity values as a function of empirical rsfMRI functional degree centrality (R^2^ = .731).


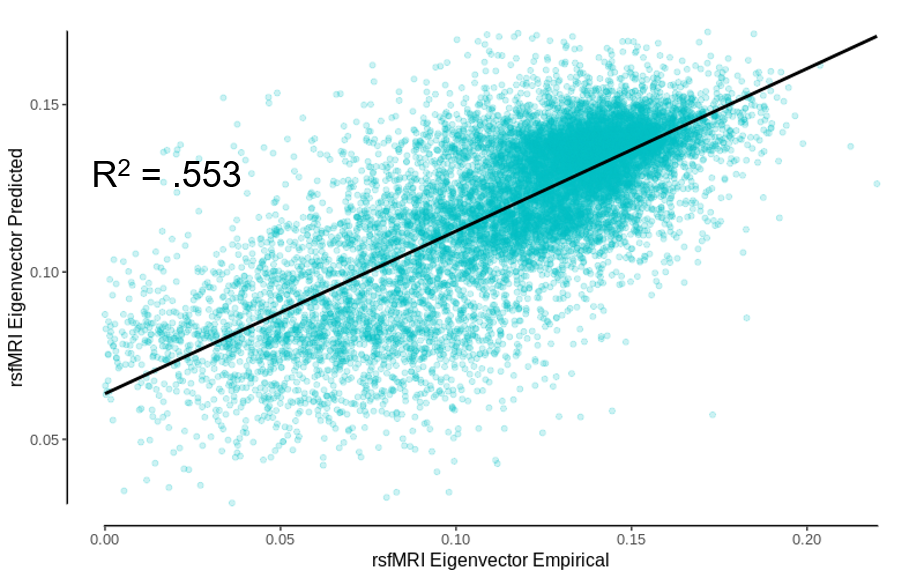


*Supplementary Figure 5*. DK atlas non-aggregated rsfMRI functional eigenvector centrality measures calculated from the predicted functional connectivity values as a function of empirical rsfMRI functional eigenvector centrality (R^2^ = .553).


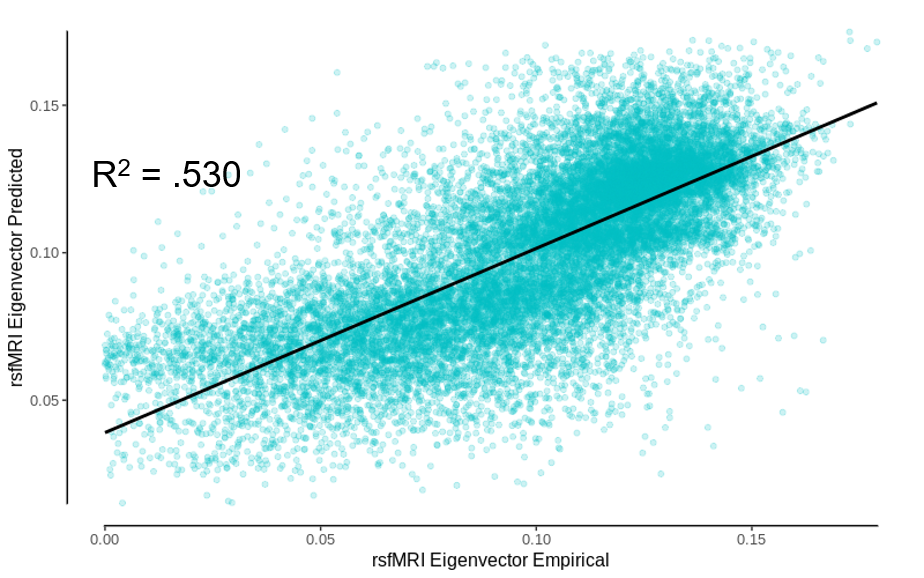


*Supplementary Figure 6*. AAL atlas non-aggregated rsfMRI functional eigenvector centrality measures calculated from the predicted functional connectivity values as a function of empirical rsfMRI functional eigenvector centrality (R^2^ = .530).


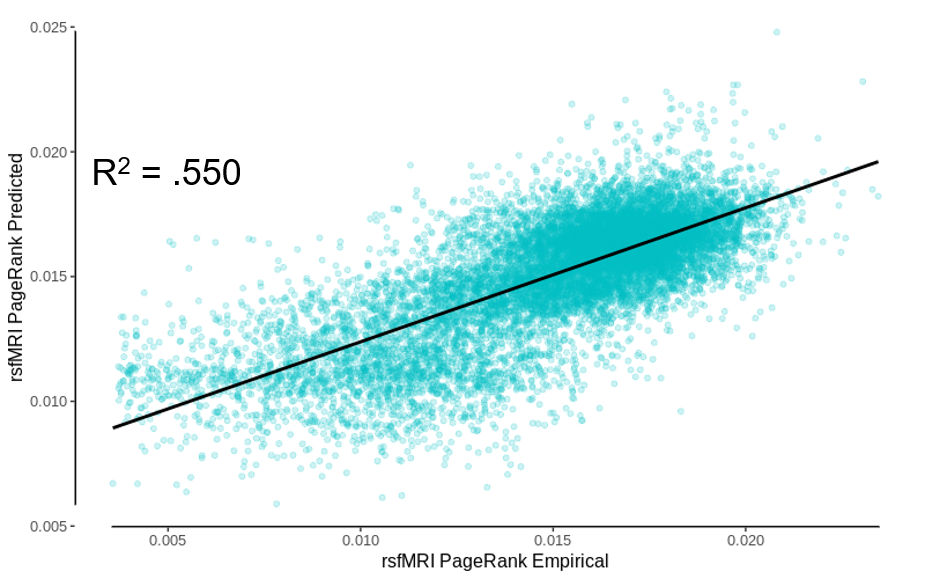


*Supplementary Figure 7*. DK atlas non-aggregated rsfMRI functional PageRank centrality measures calculated from the predicted functional connectivity values as a function of empirical rsfMRI functional PageRank centrality (R^2^ = .550).


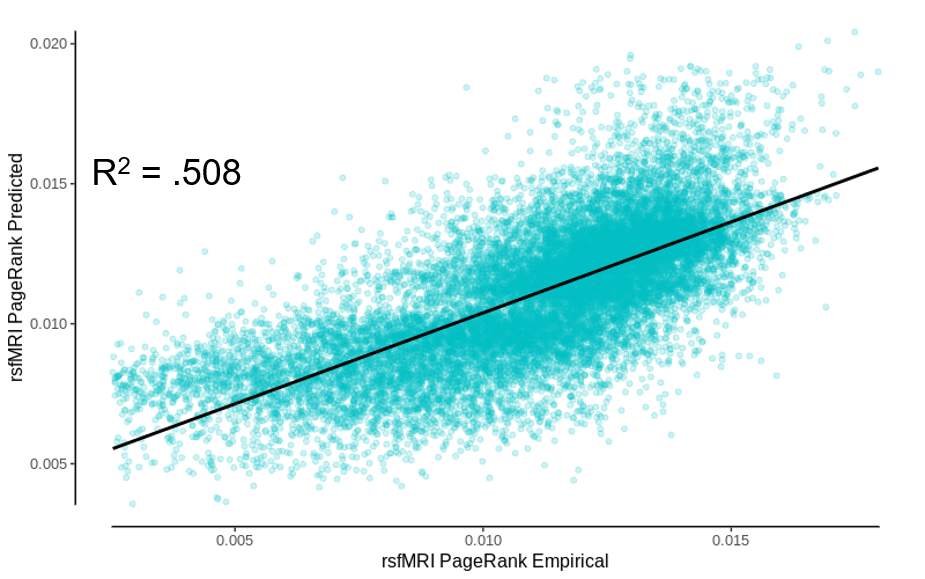


*Supplementary Figure 8*. AAL atlas non-aggregated rsfMRI functional PageRank centrality measures calculated from the predicted functional connectivity values as a function of empirical rsfMRI functional PageRank centrality (R^2^ = .508).


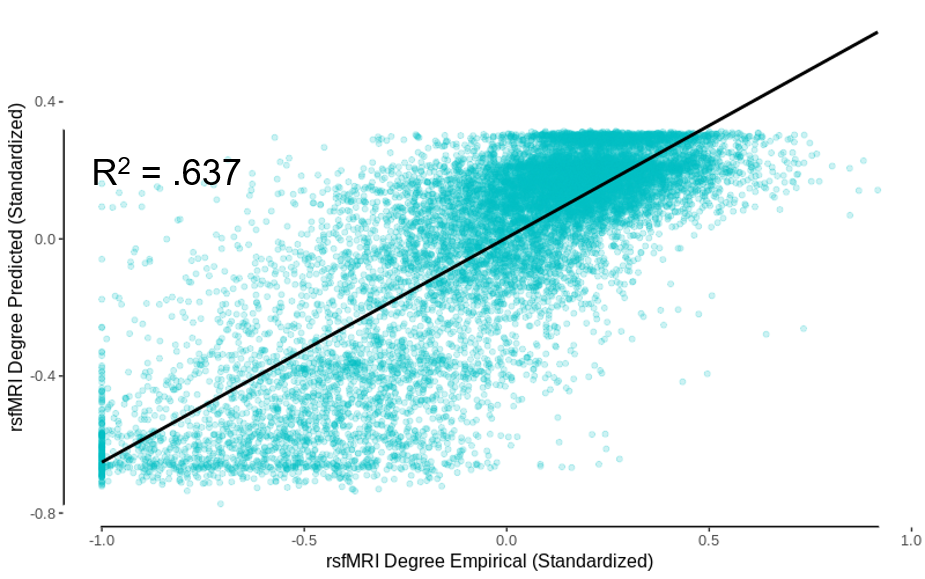


*Supplementary Figure 9*. DK atlas non-aggregated predicted rsfMRI functional connectivity degree centrality as a function of empirical rsfMRI functional degree centrality (R^2^ = .637).


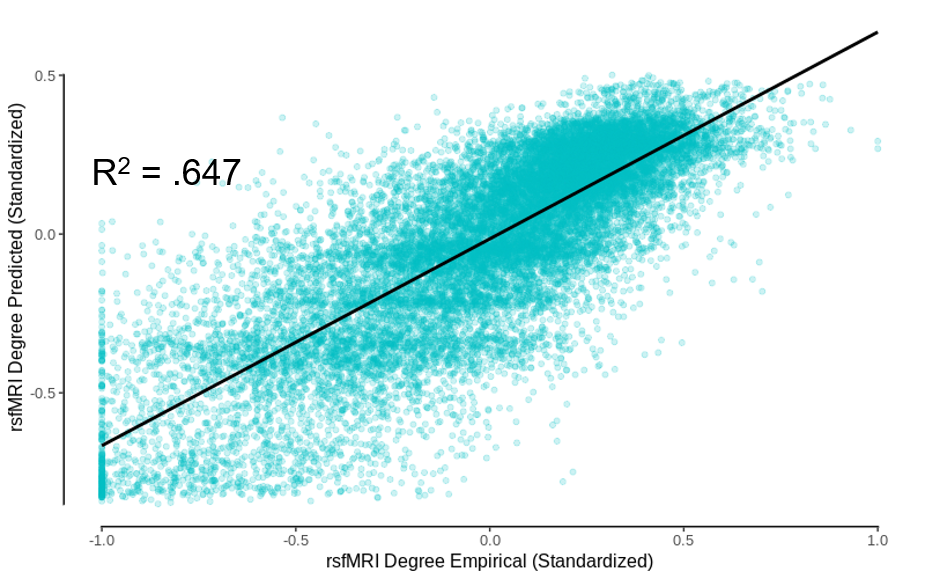


*Supplementary Figure 10*. AAL atlas non-aggregated predicted rsfMRI functional connectivity degree centrality as a function of empirical rsfMRI functional degree centrality (R^2^ = .647).


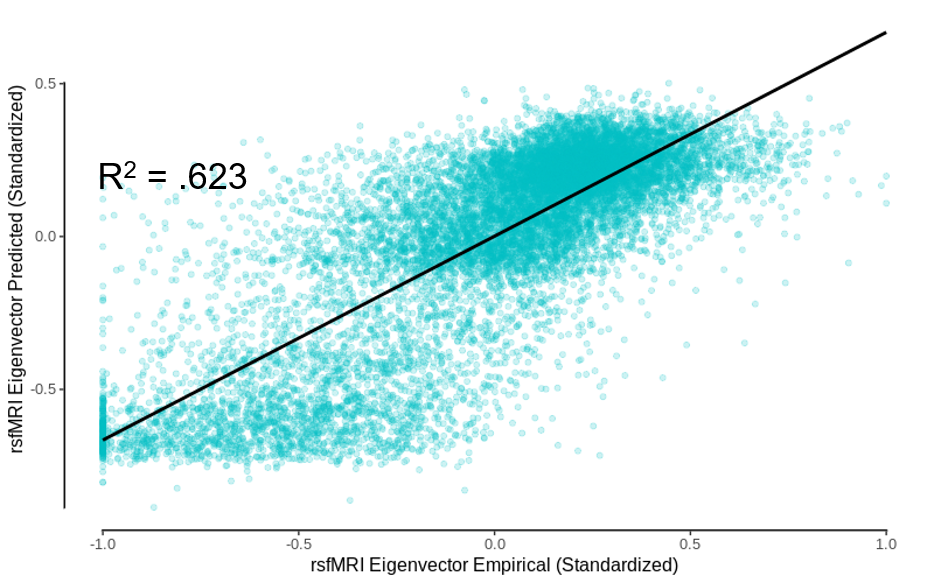


*Supplementary Figure 11*. DK atlas non-aggregated predicted rsfMRI functional connectivity eigenvector centrality as a function of empirical rsfMRI functional eigenvector centrality (R^2^ = .623).


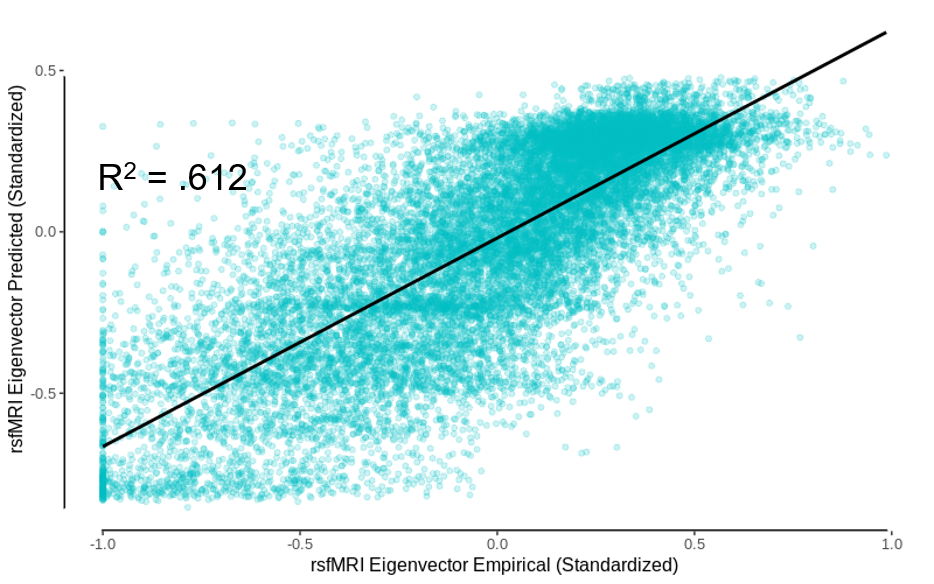


*Supplementary Figure 12*. AAL atlas non-aggregated predicted rsfMRI functional connectivity eigenvector centrality as a function of empirical rsfMRI functional eigenvector centrality (R^2^ = .612).


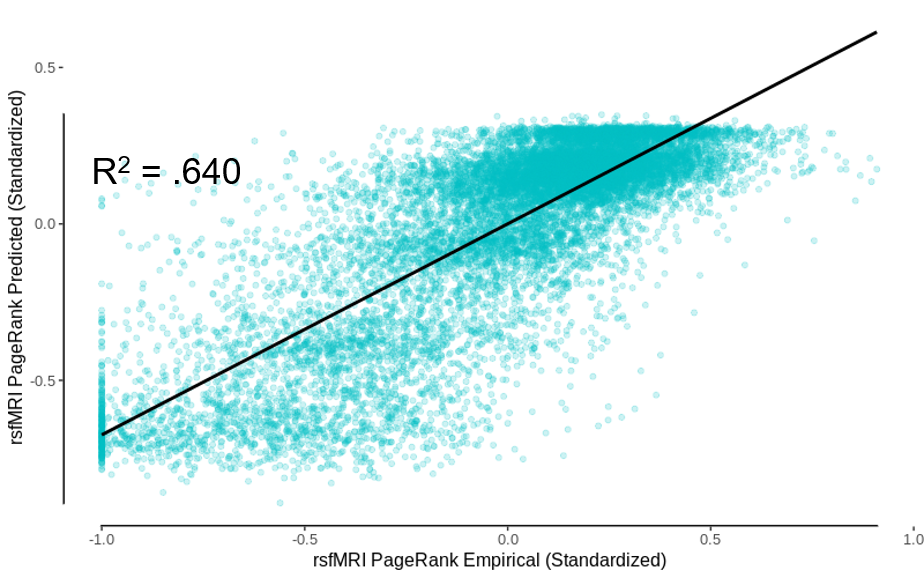


*Supplementary Figure 13*. DK atlas non-aggregated predicted rsfMRI functional connectivity PageRank centrality as a function of empirical rsfMRI functional PageRank centrality (R^2^ = .640).


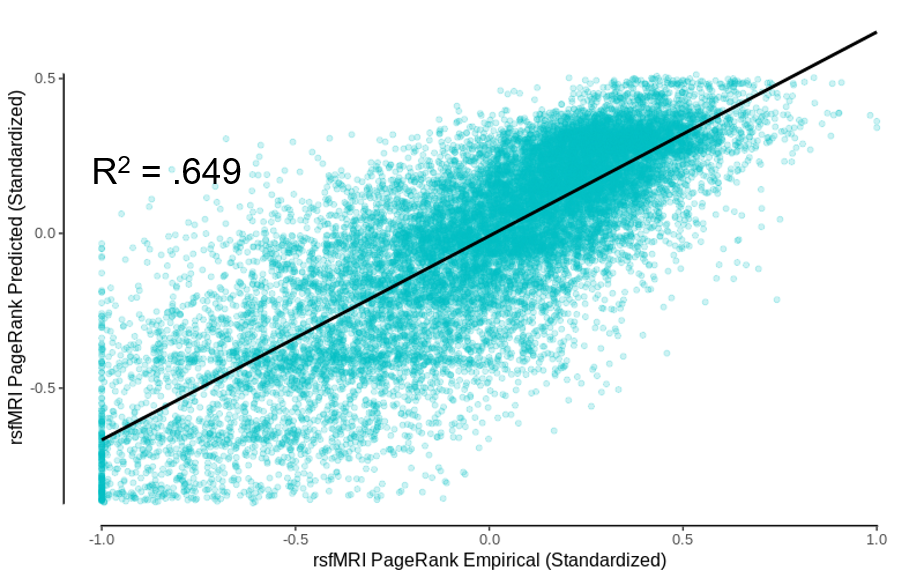


*Supplementary Figure 14*. AAL atlas non-aggregated predicted rsfMRI functional connectivity PageRank centrality as a function of empirical rsfMRI functional PageRank centrality (R^2^ = .649).
